# Asymptomatic Malaria and Helminths Coinfection and Its Association with Anemia among Primary School Children in Gedeo Zone, Southern Ethiopia: A Cross-Sectional Study

**DOI:** 10.1155/2021/7742960

**Published:** 2021-09-13

**Authors:** Feven Wudneh, Yabibal Gebeyehu, Sara Anberbir

**Affiliations:** ^1^Department of Medical Laboratory, College of Medicine and Health Sciences, Dilla University, Dilla, Ethiopia; ^2^School of Medicine, College of Medicine and Health Sciences, Dilla University, Dilla, Ethiopia

## Abstract

**Background:**

Asymptomatic malaria and helminths coinfection occurs mainly in the tropics and subtropics where poverty and sanitary practice favor its high prevalence. In the tropics, where malaria is endemic, helminths also thrive resulting in coinfection. This study aimed to access the prevalence of asymptomatic malaria and helminths coinfection and its contribution for anemia in primary school children of Gedeo Zone, Southern Ethiopia. *Methodology*. This was a cross-sectional study conducted among 413 primary school children from February to April 2020. Finger-prick blood samples were used to determine asymptomatic malaria and hemoglobin concentrations. Stool samples were collected and processed through formalin-ether concentration techniques to detect the presence of intestinal helminths. Data were double entered into Epi Data version 3.1 software and exported to SPSS version 20 for analysis. Pearson's chi-square and correlation analysis were performed as part of the statistical analyses.

**Result:**

A total of 413 primary school children aged 6 to 16 years (mean age ± SD: 10.7 ± 2.64years) were enrolled in the study. 159 (38.5%) of school children were infected with at least one of the parasitic diseases. The overall prevalence of asymptomatic malaria and intestinal helminths was 46 (11.1%) and 113 (27.3%) respectively. Asymptomatic malaria and helminths coinfection was 29 (7%). Total of 39.1% of asymptomatic malaria-infected school children were anemic, which is statistically significant (*P* < 0.05). 15.9% of helminths-infected school children were anemic, not statistically significant (*P* > 0.05). The prevalence of anemia was 12 (41.3%) among coinfected students, which is statistically significant (*P* < 0.005).

**Conclusion:**

Asymptomatic malaria and helminths coinfection affects the health status of considerable number of primary school children in the study area. Therefore, simultaneous combat against the two parasitic infections is crucial to improve health of the school children.

## 1. Introduction

Asymptomatic malaria and helminths infection are among the most prevalent diseases in sub-Saharan Africa. It is estimated that over one-third of the world's population, mainly those individuals living in the tropics and subtropics, are infected by intestinal helminths or one or more of *Plasmodium* species [1]. Both intestinal helminthic and malarial infection cause high rates of morbidity. Climatic conditions, poverty, and sanitary practices favor their high rate of prevalence in developing countries [2].

Although there is no standard definition for “asymptomatic” malaria infections, it is generally accepted to be malarial parasitemia of any density, in the absence of fever or other acute symptoms in individuals who have not received recent antimalarial treatment [3]. There are about 300–500 million incidences of malaria causing 2-3 million deaths each year in the tropical and subtropical regions of the world [4]. About 90% of these deaths occur in sub-Saharan Africa [4]. In Ethiopia, the burden of malaria continues to cause a substantial number of morbidity and mortality which accounts for most outpatient visits. Malaria has been one of the main causes of hospitalization and deaths in the country. About 60% of the population are living in the malaria-risky areas, mainly areas that lie below 2000 m above sea level. However, several pockets with microepidemiological conditions supporting malaria transmission occur in areas above this altitude. *Plasmodium falciparum* and *P*. *vivax* are the dominant parasites responsible for the majority of malaria cases in Ethiopia [5–7], and malaria is ranked as the leading communicable disease accounting for about 30% of the overall disability adjusted life years lost [8].

Intestinal helminths are also the cause of malnutrition, or aggravate existing malnutrition; through damage to the gastrointestinal mucosal epithelium and inflammation, reduced food intake, malabsorption, and increased nitrogen loss in faeces and diarrhea, malaria-helmiths coinfections further compromise the health condition of the patient [9–12].

In Ethiopia, like other developing countries, infections with the major soil-transmitted helminths, including *Ascaris lumbricoides*, *Trichuris trichiura*, Hook worm, and *Hymenolophis nana*, are widely spread with variable prevalence [13–15].

Asymptomatic malaria and helminths coinfection overlapping distribution contributes substantially to anemia [16]. Malaria in endemic regions causes anemia through mechanisms such as haemolysis, increased spleen clearance of infected and uninfected red blood cells and cytokine-induced dyserythropoesis, destruction of parasitized red blood cells, shortening of the life span of nonparasitized red blood cells, and decreased production of red blood cells in the bone marrow [2]. Mechanisms of anemia include lysis and phagocytosis of infected red blood cells while depending on the intensity of the malaria infection [17]. An asymptomatic malaria host serves as a reservoir for the malaria parasite, and asymptomatic malaria is now recognized as an important obstacle to malaria elimination [18]. Asymptomatic malaria and helminths coinfection greatly affects the socioeconomic development of communities in multiple ways. From health perspective, it affects the physical and mental wellbeing of school children, thereby leading to increased absenteeism and retarded cognitive development, and thus, learning disabilities although the literature is inconclusive [10, 19]. Most importantly, helminths complicate the clinical picture of more serious diseases such as malaria and cause anemia as a result of direct blood loss, nutritional theft, and impairment of appetite due to immunological factors.

There is evidence that *Ascaris lumbricoides*-associated vitamin A deficiency may further increase the risk of anemia in those coinfected with malaria [18]. Hookworms are the most pathogenic because of their propensity to feed on blood [20].

This parasitic disease affects the health status of the community in sub-Saharan Africa. Also, studies indicated that individuals coinfected with more than one parasite species are at risk of increased morbidity, as well as at a risk of developing frequent and more severe disease due to interactions among the infecting parasite species [21, 22]. However, how concurrent infections affect the epidemiology and/or the pathogenesis of each other remains controversial ranging from increased severity of malaria to reduced severity and incidence of malaria during helminths coinfection [23–25].

Therefore, this study aimed to assess the prevalence of intestinal helminths and asymptomatic malaria and its effects on anemia among primary school children. The results of this study could help concerned stakeholders to take action on the prevention of intestinal helminths and asymptomatic malaria.

## 2. Materials and Methods

### 2.1. Study Design and Period

A cross-sectional study was conducted from February to April 2020 in selected Dilla town and Dilla Zuria woreda primary schools.

### 2.2. Study Area

**Figure 1:**
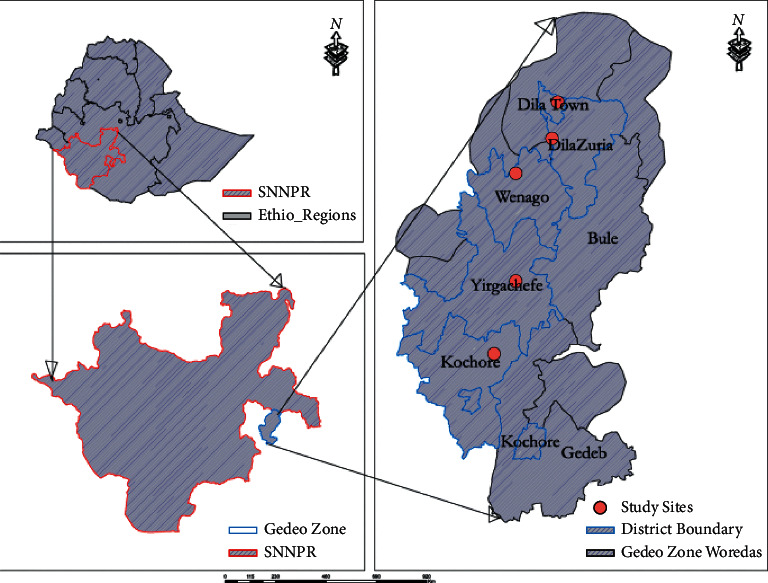
Map of Gedeo zone.

Dilla is located 360 km to the south of Addis Ababa, the capital of Ethiopia, with an area of 1123.47 sq.kms. It is located 5°53′N to 6°27′N latitude and 38°8′ to 38°30′ east longitude. Dilla town and Dilla Zuria woreda are located in Gedeo zone (Figure 1). The altitude of the zone ranges from 1268 to 2993 m above sea level (masl). The mean annual temperature is between 12.6°C and 30°C. The mean annual rainfall ranges from 1001 to 1800 mm, and the pattern is bimodal, with a short rain season between March and May accounting for 30% of total rain fall. The long rain season is between July and October accounting for more than 60% of the total rainfall.

#### 2.2.1. Study Population

All primary school children aged 5 to 16 years attending class in Dilla town and Dilla Zuria woreda and who did not take antimalarial and antihelminths drug two weeks prior to the study commencing were considered as the study population.

#### 2.2.2. Sampling Techniques

There are 31 primary schools in Dilla and Dilla Zuria woreda. Six of them were selected by simple random sampling and by considering proportional allocation: three primary schools (Kirinchaf, Dawit, and Kofe) and another three primary schools (Sisota, Aroresa, and Chichu) were selected from Dilla and Dilla Zuria woreda, respectively. Finally, representative primary school children were selected by systematic random sampling techniques from the registration list of selected schools.

#### 2.2.3. Sample Size Determination

Sample size was calculated using a single population proportion formula by taking a previous similar study conducted in Alaba kulito [12], 95% confidence interval, 5% margin of error, and 10% nonresponse rate (38 students), and a total of 417 participants were enrolled in the study.

#### 2.2.4. Data Collection and Study Sample

Sociodemographic data were collected using pretested structured questionnaires prepared in local languages (Gede'offa and Amharic). The study participants were also interviewed to obtain sociodemographic characteristics and other information (age, sex, hand wash practice after toilet use, shoe wearing habit, source of drinking water, and residence). A total of 417 primary school children were enrolled in the study, and three of them were excluded from the study since they did not fulfill the inclusion criteria. Therefore, a total of 413 primary school children participated in the study.

#### 2.2.5. Blood Film Preparation and Examination of Malaria Parasites

The clean slides were labeled with patient identification number, date, and time of collection at the frosted end. The blood sample was collected from the middle or ring finger after cleaning with a 70% alcohol-moistened swab and dried with a piece of dry cotton after pricked using a disposable blood lancet. Through wiping off the first drop of blood, 6 *μ*l of blood for thick and 2 *μ*l of blood for thin blood films were collected [22, 26]; then; slides were air-dried and fixed by using absolute methanol for 30 seconds before staining. Staining was carried out using 10% Giemsa solution for 10 minutes. The stained slides were rinsed with water, air-dried, and finally, examined using a microscope. To avoid false positive and negative result, microscopic examination was performed by two experts while discrepancies were resolved by a third opinion [27, 28]. Smears were reported as negative after observing at least 100 fields using high-power fields (oil immersion).

#### 2.2.6. Formalin-Ether Concentration Method

A stool sample was collected from each participant using a clean, leak-proof, and sterile stool cup and preserved using 10% formalin and then processed by the formalin-ether concentration procedure; 2 ml of preserved stool was added in a clean conical centrifuge tube containing 7 ml of 10% formol water. The suspension was filtered through a sieve into a 15 ml conical centrifuge tube. Then, 4 ml of diethyl ether was added to the formalin solution and the content was centrifuged at 300 rpm for 1 minute. The supernatant was discarded, and stool smears were prepared on sterile slides from the sediment and then three slides were prepared for each participant [22, 29]. Finally, the slides were microscopically examined by two experts while discrepancies were resolved by a third opinion under a magnification power of 10x and 40x objective to identify intestinal helminths.

#### 2.2.7. Hemoglobin Concentration

Hemoglobin concentration was determined using a Hemocue Hb 201 analyser [30, 31]. Anemia classification was interpreted by the WHO anemia classification. According to the WHO guidelines, for children 5–11 years of age, hemoglobin levels of 11–11.4 g/dl were considered as mild, 7–10.9 g/dl moderate, and <7 g/dl severe anemia and for children 12–16 years of age, the hemoglobin level 11–11.9 g/dl was considered as mild, 7–10.9 g/dl moderate, and <7 g/dl severe anemia [32].

#### 2.2.8. Data Quality Control

To keep the quality of data, on-site training was given for data collectors on how to collect sociodemographic data and other associated factors. Data were checked for completeness before and after entry. Reagent and instrument quality was also checked for expiry dates and any functional problems.

The slides were microscopically examined by two experts, while discrepancies were resolved by a third opinion for quality control. Similarly, quality control was performed on hemoglobin measures, Hemocue Hb 201 + analyser and *Mission*® Hemoglobin Plus Hb (an automated haematology analyser).

### 2.3. Statistical Analysis

The data were double entered to EpiData software and exported to SPSS version 20 software for analysis. Descriptive statistics was performed for sociodemographic characteristics. Pearson's chi-square and multivariate logistic regression association were also performed to assess the association between malaria/helminths coinfection and anemia. Statistical significance were considered if *P* < 0.05.

### 2.4. Ethical Consideration

Ethical clearance was received from the research and ethical review committee of Dilla University. Before data collection, the objectives of the study were explained to the study participants, parents, and the school community. Written informed consent was obtained from partners or the legal guardians. Children infected with any of the parasites were referred to the nearby health institution for treatment. All information obtained from the study participants was coded to keep confidentiality.

## 3. Results

### 3.1. Sociodemographic Characteristics

A total number of 413 participants were enrolled in the study. About 322/413 (78%) of the study participants were between the age range of 10–15 years. Two hundred and twenty-seven study participants (55%) were males, 132 (32%) used bed net, 362 (87.5%) washed their hand before and after toilet use, and 305 (73.8%) and 108 (26.2%) of study participants wore shoe always and sometimes, respectively. About 374 (90.3%) used toilet, and the rest 40 (9.7%) used open space for defecation. The source of drinking water as pipe, well, and river was 322 (78%), 84 (20.3%), and 6 (1.5%), respectively. Two hundred and seventeen (51.8%) of them were living in urban areas (Table 1).

#### 3.1.1. Prevalence of Asymptomatic Malaria

**Figure 2:**
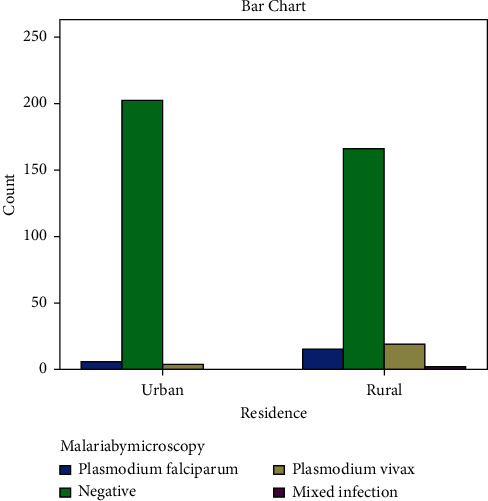
The prevalence of asymptomatic malaria among urban and rural residence of primary school children in Gedeo zone, Southern Ethiopia.

The overall prevalence of asymptomatic malaria was 46 (11.1%), 21 (5.1%) *Plasmodium falciparum*, 23 (5.6%) *Plasmodium vivax,* and 2 (0.4%) mixed infections. The majority, 29 (7%) of malaria-infected students were in the age group of 10–14 years. The prevalence of asymptomatic malaria among males and females was 21 (5.1%) and 25 (6%), respectively (Table 2), and higher prevalence was observed among rural students (Figure 2).

#### 3.1.2. Prevalence of Intestinal Helminths Infection

Out of 413 children, 113 (27.3%) were infected by at least one intestinal helminths. The prevalence of intestinal helminthic infection was higher, 85 (20.5%), among the age group of 10–14 years. *Ascaris lumbricoides* was the most prevalent with 57 (13.8) (Table 3).

#### 3.1.3. Asymptomatic Malaria and Helminths Coinfection

The prevalence of asymptomatic malaria and helminths coinfection was 29 (7%). The magnitude of *Ascaris lumbricoides*, Hook worm, and *Taenia* species among malaria-infected students was 18 (4.3%), 10 (2.5%), and 1 (0.2%), respectively (Table 4).

Students of 10–14 years were the most affected group, 20 (69%). *Ascaris lumbricoides/plasmodium vivax* and *Ascaris lumbricoides/Plasmodium falciparum* takes a major share. The prevalence of coinfection among males and females was 11 (38%) and 18 (62%), respectively (Table 5).

#### 3.1.4. Haemoglobin Measurement

From 413 participants tested for anemia, 45 (10.9%) were found to be anemic according to the WHO classification. The prevalence of anemia was significant among the age group of 5–9, *P* value 0.004, and there was no significant association observed among sex groups, *P* value 0.37 (Table 6).

Among 46 (11.1%) asymptomatic malaria-infected students, 18 (39.1%) were anemic, which is statistically significant (*P*=0.037). However, among 113 (27.3%) students infected with helminths, 18 (15.9%) were anemic, not statistically significant (*P*=0.58), and among 29 (7%) malaria-helminths coinfected students, 12 (41.3%) were anemic, which is statistically significant (*P*=0.003) (Table 7).

## 4. Discussion

The overlapping distribution of asymptomatic malaria and intestinal helminths coinfection is the main cause of anemia. In this study, we have assessed the magnitude of asymptomatic malaria and helminths coinfection, as well as its effect on the hemoglobin level, among primary school children of Gedeo Zone, Southern Ethiopia.

The prevalence of asymptomatic malaria among primary school children was 46 (11.1%). The magnitude of *Plasmodium vivax*, *Plasmodium falciparum,* and mixed infection was 23 (5.6%), 21 (5.1%), and 2 (0.5%), respectively. This finding is higher than that of the study conducted in Southern Ethiopia, 3.6% [33], and Democratic Republic, 6.3% [17], and lower than that of the study reported from Ethiopia, 18.3% [22], and Côte d' Ivoire, 50.3% [34]. The difference might be due to environmental variation, level of bed net distribution, seasonal variation, or the geographical difference of study participants.

The prevalence of asymptomatic malaria decreased when the age of participants increased. The reason might be related with the development of protective immunity by repeated infections as well as acquisition of knowledge on malaria prevention and control mechanisms. A significant number of asymptomatic malaria cases were also observed in rural areas, which might be associated with suboptimal use of bed net. On the other hand, the prevalence of helminths was 113 (27.6%) and it was lower than that of studies conducted in Southern Ethiopia, 69% [34], and Northwest Ethiopia, 49% [35], and higher than that of the study conducted in North West Tigray, 12.7% [36]. These might be related with school-based mass deworming campaigns conducted in the study area.

The prevalence of asymptomatic malaria and helminths coinfection was 29 (7%). The prevalence of *Ascaris lumbricoides* coinfection with *Plasmodium falciparum* and *Plasmodium vivax* was 8 (1.9%) and 10 (2.4%), respectively. The prevalence Hookworm coinfection with *Plasmodium falciparum and Plasmodium vivax* was 4 (1%) and 6 (1.45%), respectively. This finding is lower than that of the study conducted in Southwest Nigeria [11], Côte d' Ivoire [34], and Ghana [16] and larger than that of the study in Nigeria [37]. This could be linked with environmental contamination, poor hand washing practice, insufficient latrine constriction, open air defecation, and small-scale distribution of bed net. A significant association of asymptomatic malaria and hook worm coinfection was observed. The coinfection had various underlying mechanisms including environmental, socioeconomic, and behavioural factors or due to involvement of immunological mechanisms which might lead to increased susceptibility of helminths-infected individuals for malaria infection.

The prevalence of anemia among asymptomatic malaria and helminths coinfected students was 41.3%. This findings was higher than that of the study conducted in Northwest Ethiopia, 10.9% [2], Southwest Nigeria, 34.4 [11], and Tanzania, 19.8 [29]. The result showed that the level of anemia among coinfected students was significantly higher. This might be associated with the blood loss mainly caused by the destruction of RBC and hook worm infection similar with the finding of the study conducted by Garcia LS [38].

## 5. Conclusions

Asymptomatic malaria and helminths coinfection was observed in the study area, and the prevalence of anemia was significantly associated with coinfection. Therefore, combined and integrated interventional measures should be taken including health education for school children about personal and environmental hygiene practices, use of bed net, encouraging footwear practice, and use of latrine.

## Figures and Tables

**Table 1:** Sociodemographic characteristics of primary school children in Gedeo zone, Ethiopia.

Variables	Category	*N*	Percent (%)
Age	5–9	61	14.8
	10–14	322	78
	>15	30	7.2

Sex	M	227	55
	F	186	45

Residence	Urban	217	52.5
	Rural	196	47.5

Religion	Orthodox	98	23.6
	Muslim	33	8
	Protestant	282	68.4

Bed net use	Yes	132	32
	No	281	68

Source of drinking water	Pipe	322	78
	Well	84	20.3
	Underground	9	1.7

Hand washing practice before meal	Yes	362	87.5
	No	51	12.5

Habit of wearing shoe	Always	305	73.8
	Sometimes	108	26.2

Latrine use	Toilet	374	90.3
	Open space	40	9.7

**Table 2:** The prevalence of asymptomatic malaria among age and sex groups of primary school children in Gedeo zone, Ethiopia.

Variables		Asymptomatic malaria by microscopy *N* (%)		
	*Plasmodium falciparum*	*Plasmodium vivax*	Mixed infection	Total
Age				
5–9	6 (1.5%)	7 (1.7)	1 (0.2)	14 (3.4)
10–14	14 (3.4)	14 (3.4)	1 (0.2)	29 (7)
>15	1 (0.2)	2 (0.5)	0	3 (0.7)

Sex				
M	9 (2.2)	11 (2.7)	1 (0.2)	21 (5.1)
F	12 (2.9)	12 (2.9)	1 (0.2)	25 (6)

Total	21 (5.1)	23 (5.6)	2 (0.4)	46 (11.1)

**Table 3:** Prevalence of intestinal helminths among age and sex groups of primary school children in Gedeo zone, Ethiopia.

	Prevalence of helminths *N* (%)					Total	*P* value
	*Ascaris lumbricoides*	*Tricuris tricura*	Hook worm	*H*. *nana*	*Taenia* spp.		
Sex							
M	41 (9.9)	4 (1)	6 (1.5)	7 (1.7)	1 (0.2)	59 (14.3)	0.194
F	36 (8.7)	4 (1)	9 (2.1)	5 (1.2)	0	54 (13.1)	

Age							
5–9	14 (3.4)	0	5 (1.2)	1 (0.2)	0	20 (4.8)	0.012
10–14	57 (13.8)	8 (2)	10 (2.4)	9 (2.1)	1 (0.2)	85 (20.5)	
>15	6 (1.4)	0	0	2 (0.5)	0	8 (1.93)	

Total	77 (18.6)	8 (2)	15 (3.6)	12 (2.9)	1 (0.2)	113 (27.6)	

**Table 4:** Prevalence of asymptomatic malaria and helminths coinfection among primary school children in Gedeo zone, Southern Ethiopia.

Type of helminths	Result	Asymptomatic malaria infection, *N* (%)		OR (95% CI)	*P* value
		Positive	Negative		
*A. lumbricoides*	Positive	18 (4.3)	59 (14.3)	1.6 (0.7–3.6)	0.2
	Negative	28 (6.7)	308 (74.6)		

Hook worm	Positive	10 (2.5)	5 (1.2)	6 (1.04–34.54)	0.045
	Negative	36 (8.7)	362 (87.7)		

*Taenia* spp.	Positive	1 (0.2)	0		
	Negative	45 (10.9)	367 (88.9)		

**Table 5:** Prevalence of asymptomatic malaria and helminths coinfection among the age and sex group of primary school children in Gedeo zone, Southern Ethiopia.

Variables	Asymptomatic malaria and helminths coinfection by age and sex, *N* (%)					
	AL + PF	AL + PV	HW + PF	HW + PV	PV + *Taenia*	Total
Age						
5–9	1 (3.45)	3 (10.3)	1 (3.45)	1 (3.54)	1 (3.45)	7 (24)
10–14	6 (20.6)	6 (20.6)	3 (10.3)	5 (17.2)	0	20 (69)
>15	1 (3.45)	1 (3.45)	0	0	0	2 (7)

Sex						
M	2 (6.9)	4 (13.8)	1 (3.45)	3 (10.34)	1 (3.45)	11 (38)
F	6 (20.7)	6 (20.7)	3 (10.3)	3 (10.34)		18 (62)

Total	8 (27.5)	10 (34.5)	4 (13.8)	6 (20.7)	1 (3.45)	29 (100)

PF = *P*. *falciparum*; PV = *P*. *vivax*; HW = Hook worm; AL = *Ascaris lumbricoides*.

**Table 6:** Anemia level among the age and sex group of primary school children in Gedeo zone, Southern Ethiopia.

Variables	Anemia, *N* (%)	*P* value	AOR	95% confidence interval	
				Lower bound	Upper bound
Age					
5–9	12 (2.9)	0.004	13.9	2.4	82.8
10–14	31 (7.5)		2.3	0.5	10.9
>15	2 (0.5)				

Sex					
M	24 (5.8)	0.14	1.3	0.6	2.7
F	21 (5.1)				

Total	45 (10.9)				

**Table 7:** Prevalence of anemia among asymptomatic malaria, helminths, and coinfected primary school children in Gedeo zone, Southern Ethiopia.

Type of infection	Anemia			
	Absent (%)	Present (%)	*P* value	Total, *N* (%)
Asymptomatic malaria	27 (60)	18 (40)	0.037	45 (10.9)
Intestinal helminths	95 (84)	18 (15.9)	0.58	113 (27.4)
Coinfection	17 (58.6)	12 (41.3)	0.003	29 (7)

## Data Availability

The data underlying this article are available in the article and in the supplementary materials.

## Abbreviations

RBCs: Red blood cells
PF: *Plasmodium falciparum*
PV: *Plasmodium vivax*
STH: Soil-transmitted helminths
WHO: World Health Organization.

## Additional Points

In the currents study, we did not use advanced diagnostic techniques such as PCR and other advanced parasitological techniques. PCR is important to identify the gene which makes malaria asymptomatic and increase diagnostic accuracy and sensitivity. The other limitation of this study was we did not rule out other possible causes of anemia such as malnutrition.

## Supplementary Materials

Supplementary Materials The data of this article are given.
