# Supplementary material for: Asymptomatic Malaria and Helminths Coinfection and Its Association with Anemia among Primary School Children in Gedeo Zone, Southern Ethiopia: A Cross-Sectional Study
Source: J Trop Med. 2021 Sep 13;2021:7742960. doi: 10.1155/2021/7742960 (PMC8452447; doi:10.1155/2021/7742960)
Supplement: Supplementary Materials — The data of this article are given. [file 7742960.f1.pdf]

DECLARATION

Ethical Clearance

ዲላ ዩኒቨርስቲ
ጤናና ህክምና ሳይንስ ኮሌጅ
የምርምር ሥነ-ምግባር ግምገማ ቦርድ

Dilla University
College of Medical, Health Sciences
Institutional Review Board (IRB)

Ref.No: 015/2019
Date: 26/12/2019

To: Prof./Dr./Mr./Mrs./**Mss.** Feven wudneh
Address of the researcher email fevenwudneh@yahoo.com
Subject: **Issuance of Ethical Clearance**

Protocol Unique number: 010/19-10
Study Site: Dilla University referral hospital

Dear Researcher:-

The Institutional review Board (IRB) of Dilla University, College of Health Sciences and Medicine has critically reviewed your research proposal entitled "**Prevalence of Asymptomatic Malaria and Helminthes Co-Infection and Its Association with Anemia among Primary School Children in Gedeo Zone, Southern Ethiopia, 2019.**" for ethical soundness. Accordingly, your study was declared to be ethically sound and the IRB **approved** your study for implementation. Any change in the protocol methodology should be in consultation with IRB. The IRB also request you to follow the recommended research ethics protocol and do your best not to harm your study subjects.

DO NO HARM!!

Validity period of the approval Dec 26/2019 to Dec26/2020

Signature: [illegible]
Chairperson
Date: 26/12/19

Signature: [illegible]
Secretary
Date: 26/12/19

CC.
- Institutional Review Board (IRB)
  D/U/C/M/H/S/R/Hospital

P.O.Box Tel Fax E-Mail/web

Consent to participate

I confirm that I will give consent for my child to participate in the study with a clear understanding of the objectives and conditions of the study .I have also assured that my child can withdraw at any time without penalty or loss of benefits. The proposal has been explained for me and my child in the language I understand.

Participant ID--------------------------------- Signature: ------------Date ---------------

Name of person obtaining consent------------------- Signature ------------Date --------------

Pictures took when conducting the research

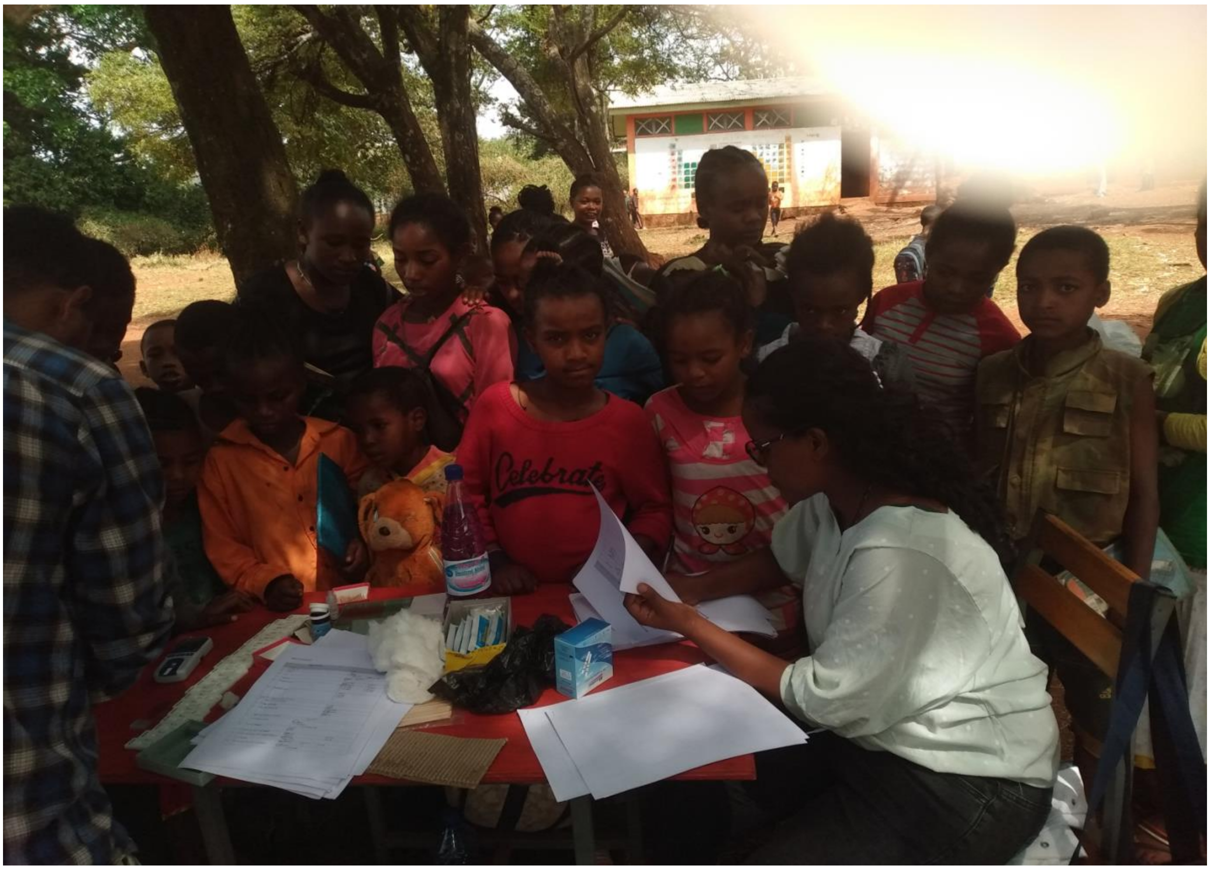

During data collection

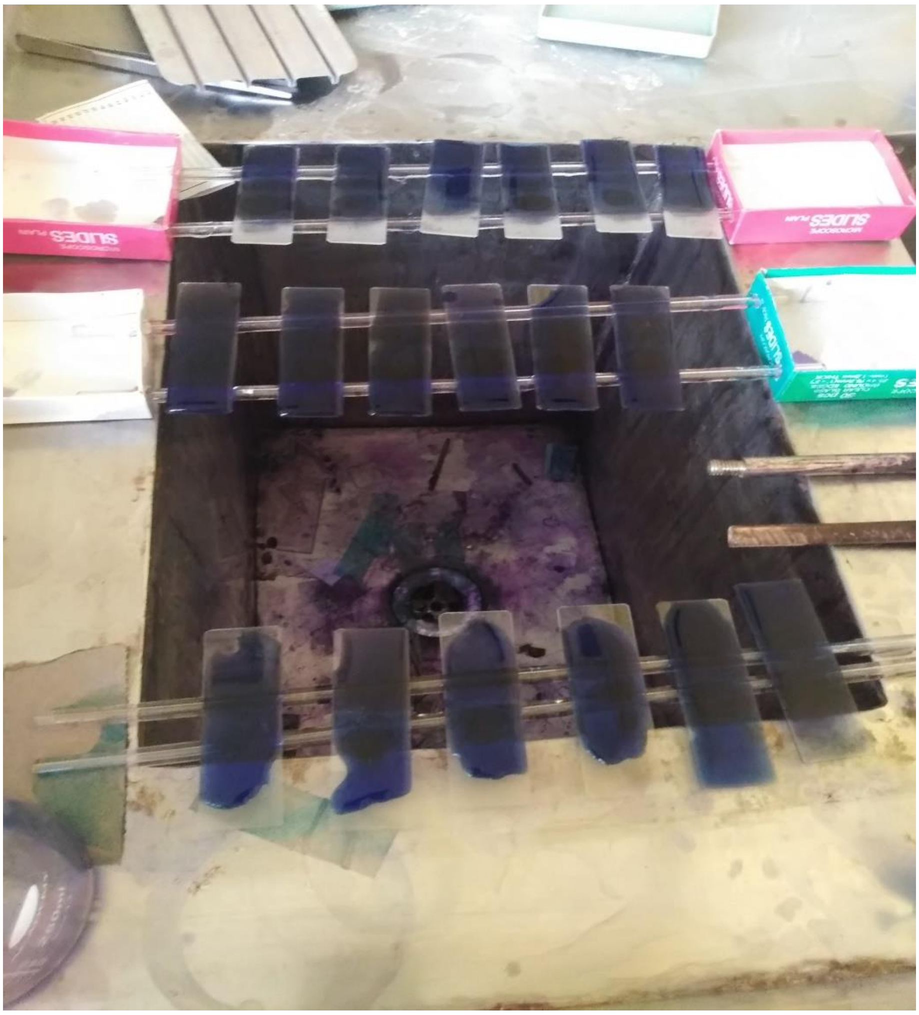

Staining with Giemsa

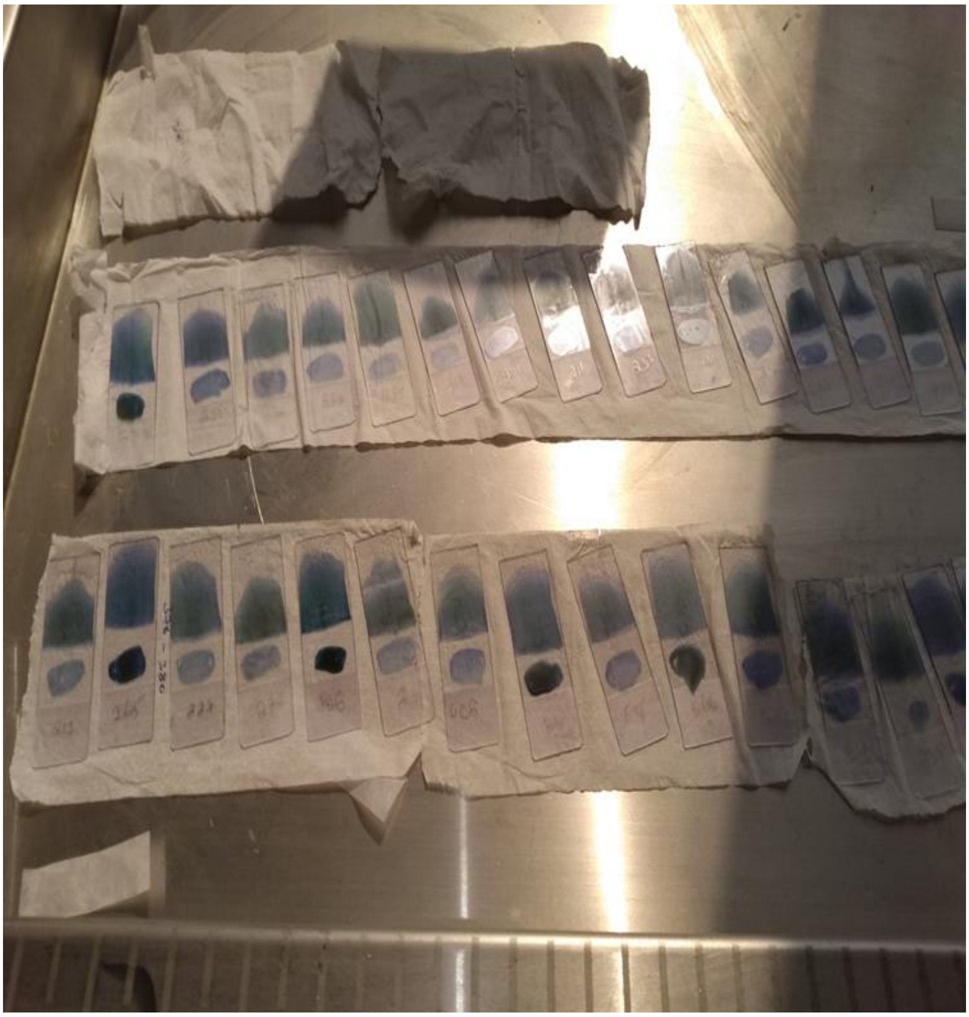

Thick and thin blood film

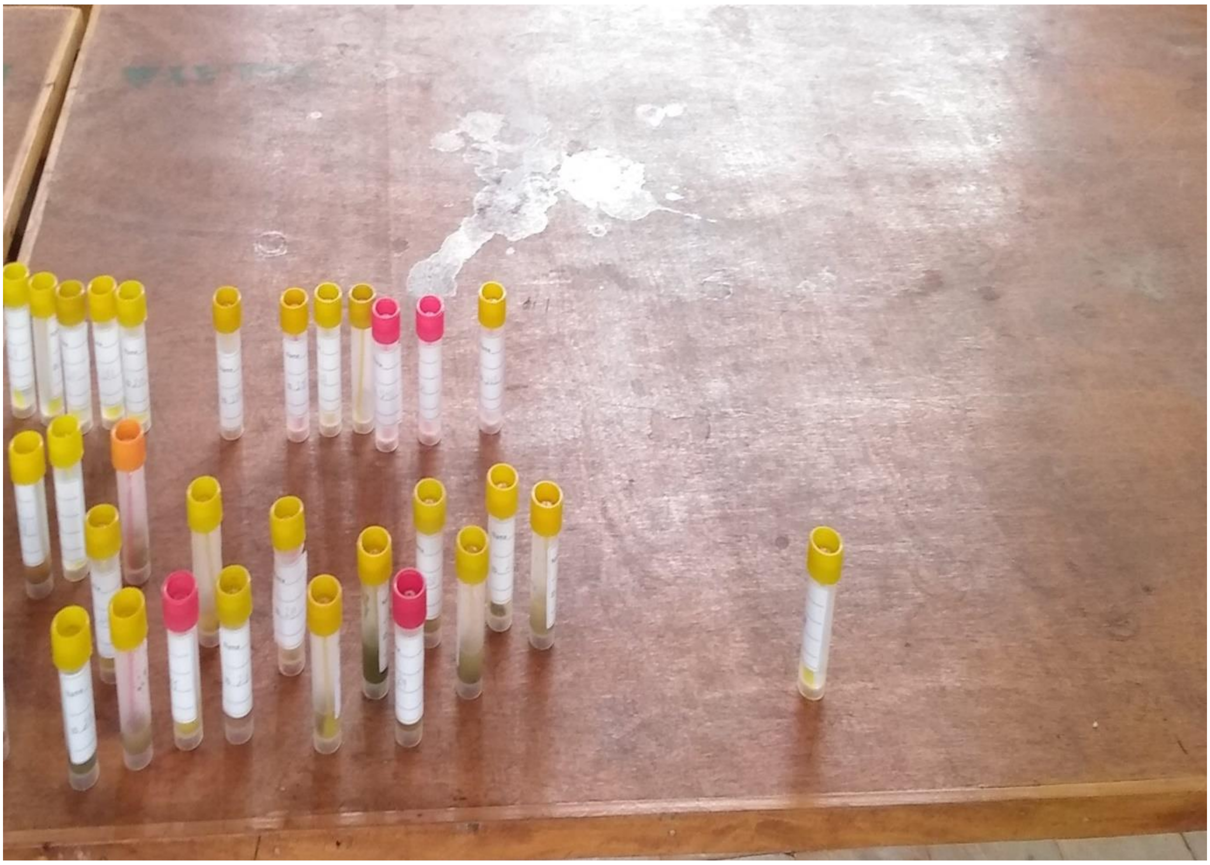

Collected stool preserved in 10%formaline
